# Structure and properties of the egg mass of the ommastrephid squid *Todarodes pacificus*

**DOI:** 10.1371/journal.pone.0182261

**Published:** 2017-08-02

**Authors:** Pandey Puneeta, Dharmamony Vijai, Jun Yamamoto, Kohsuke Adachi, Yoshiki Kato, Yasunori Sakurai

**Affiliations:** 1 Department of Marine Bioresources and Ecology, School of Fisheries Sciences, Hokkaido University, Hakodate, Japan; 2 Field Science Center for Northern Biosphere, Hokkaido University, Hakodate, Japan; 3 Laboratory of Aquatic Product Utilization, Graduate School of Agriculture, Kochi University, Monobeotsu 200, Nankoku, Kochi, Japan; 4 Marine Fisheries Research and Development Center (JAMARC), Japan Fisheries Research and Education Agency, Yokohama-city, Kanagawa, Japan; University of Shiga Prefecture, JAPAN

## Abstract

The Japanese flying squid, *Todarodes pacificus*, is thought to spawn neutrally buoyant egg masses that retain a specific location in the water column by floating at the interface between water layers of slightly different densities. It is important to understand the physical process that determines the vertical distribution of the egg masses to predict their horizontal drift in relation to embryo survival and subsequent recruitment. Here, mesocosm experiments were conducted in a 300 m^3^ tank by creating a thermally stratified (17–22°C) water column to obtain egg masses. A cage net methodology was developed to sustain egg masses for detailed observation. We measured the density of the egg masses of *T*. *pacificus*, and used this information to infer the vertical distribution patterns of the egg masses at the spawning grounds (Tsushima Strait, Japan). When measured separately, the density of the outer jelly of each egg mass was 2.7 σ units higher than that of the surrounding water. The outer jelly and the specific gravity of embedded individual eggs (~1.10) cause the egg masses to have very slight negative buoyancy relative to the water in which they are formed. Analysis of the vertical profile of the spawning ground showed that water density (σ_θ_) increased sharply at ~30 m depth; thus, egg masses might settle above the pycnocline layer. In conclusion, we suggest that *T*. *pacificus* egg masses might retain their location in the water column by floating at the interface between water layers of slightly different densities, which happen to be above the pycnocline layer (actual depth varies seasonally/annually) in the Tsushima Strait between Korea and Japan.

## Introduction

Following egg maturation, the eggs of female squids belonging to the family Ommastrephidae accumulate in paired oviducts [1]. At spawning, the eggs are coated with a secretion from the paired oviducal glands, while the paired nidamental glands secrete jelly material that forms fragile gelatinous globes that encase the egg masses [1–4]. The neutrally buoyant egg masses are thought to float in/above the pycnocline layer [1,5].

For many years, detailed investigations on the egg masses of ommastrephids were not possible, because of their occurring at inaccessible depths [6]. Consequently, because of a lack of appropriate pelagic sampling gear, direct observations could not be made [2]. Captive experiments [4, 7–14] and observations under natural conditions [12, 15–17] have substantially advanced our knowledge on egg mass characteristics. However, information related to the physical properties of ommastrephid egg masses remains limited to the study conducted by O’Dor and Balch [9]. The researchers conducted captive experiments on the egg masses of the ommastrephid *Illex illecebrosus* and reported that the initial density of the egg mass was 0.03 σ_t_ units higher than the water from which it was collected. This difference made the egg mass slightly negatively buoyant relative to the water in which it was spawned [9]. Since density depends on temperature and salinity, the subsequent sinking and settling depth (isopycnic level) of the egg mass should be determined by the rate of temperature and ionic equilibrium [9].

Chemically, the outer jelly of the egg mass is composed of a mucosubstance secreted by the nidamental gland [18]. The water-soluble mucin-type glycoprotein component of the mucosubstance is thought to generate the density and viscosity of the outer jelly of the egg mass [18–21]. The jelly also acts as a physical barrier between the eggs and the surrounding water, protecting the eggs from infestation [13,14,18].

The interior portion of the egg mass where eggs are embedded homogenously is a mucous matrix that is predominantly formed from water and an oviducal gland secretion [13,14,20]. The salt-soluble components in the oviducal gland secretion induce chorion expansion and perivitelline space formation, which are necessary for the normal development of the squid embryo [22–24].

*Todarodes pacificus* is a commercially and ecologically important ommastrephid species distributed around Japan [25,26]. Their nerito-oceanic distribution, and the relatively low effort to collect and maintain them live in land-based laboratories, made them an excellent model animal in several experiments focused on egg masses [4,13,14,20,27]. Following the generalized pattern for any ommastrephid squid, the “reproductive hypothesis” proposed for *T*. *pacificus* by Sakurai [28] and Sakurai et al. [5,29] states that females spawn in surface waters. Due to the difference in the density of the spawned egg masses with the surrounding water, the egg masses sink until reaching a buoyancy depth above the pycnocline layer. The number of days from fertilization to hatching is highly dependent on the prevailing temperature [30]. At 20°C, artificially fertilized eggs takes ~ 4 days to hatch [31]. After hatching, the offspring (hatchlings) swim to the surface and are transported to the respective feeding grounds by the currents. Laboratory experiments have shown that the optimum temperature range required for the survival and swimming activity of *T*. *pacificus* hatchlings ranges between 19.5 and 23°C [32].

It is important to understand the physical process that determines the vertical distribution of egg masses to predict their horizontal drift in relation to embryo survival and subsequent recruitment [33]. In our previous cycle of experiments [13], we observed the spawning behavior of the Japanese flying squid and how the thermocline helps to sustain the egg masses in the water column. The present study was designed to investigate the structure and durability of the egg masses, including how they function to prevent infestation, the physical properties, and the role of jelly during embryo development in the egg mass. The fate of the egg mass jelly during and after embryo development was also assessed. The properties of the egg masses observed *in vitro* were related to seawater parameters in the natural spawning grounds of the Japanese flying squid.

## Materials and methods

### Obtaining egg masses

Egg masses were obtained from captive mature *T*. *pacificus* females (autumn cohort) that had been maintained in a large experimental tank (10 m [length] × 5 m [width] × 6 m [height]; volume = 300 m^3^) located at Hakodate Research Center for Fisheries and Oceans (HRCFO), Japan, from September to October 2015. For details on tank parameters, live squid maintenance, and experiments, see Puneeta et al. [13,34]. The depth of seawater in the tank was maintained at 4.5 m and the temperature ranged from 17°C (bottom) to 22°C (top) in the water column, to create a thermocline necessary for sustaining the egg mass [13].

### Collection of broken egg masses by SCUBA diving

**Fig 1:**
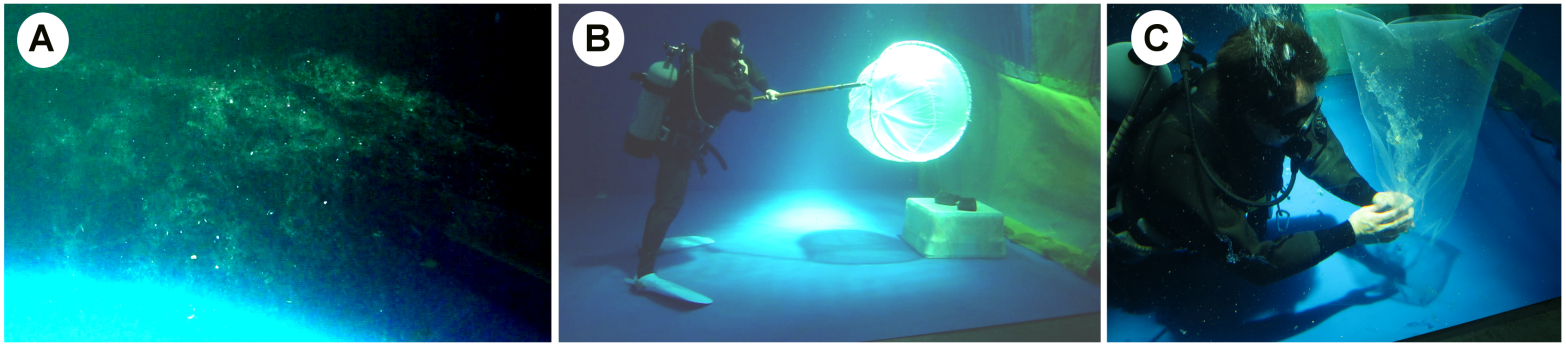
Collection of broken egg masses by SCUBA diving. (A) Egg mass fragments. (B) Collection by scoop net. (C) Collection by polythene bag.

At the beginning of the experiment, water turnover was set at 15 m^3^ h^−1^ to provide continuous, fresh seawater. The turnover volume created a circulation speed that was catastrophic to egg masses that had just been spawned. The egg masses (n = 2) that spawned during this period (one-day interval) disintegrated and floated in the mid-water as gelatinous fragments. These gelatinous fragments were collected gently by a SCUBA diver (Fig 1) to test for the presence of viable embryos and infestation. Subsequently, we reduced the circulation speed to a turnover rate of 5 m^3^ h^−1^.

### Collection and maintenance of egg masses in a cage net

**Fig 2:**
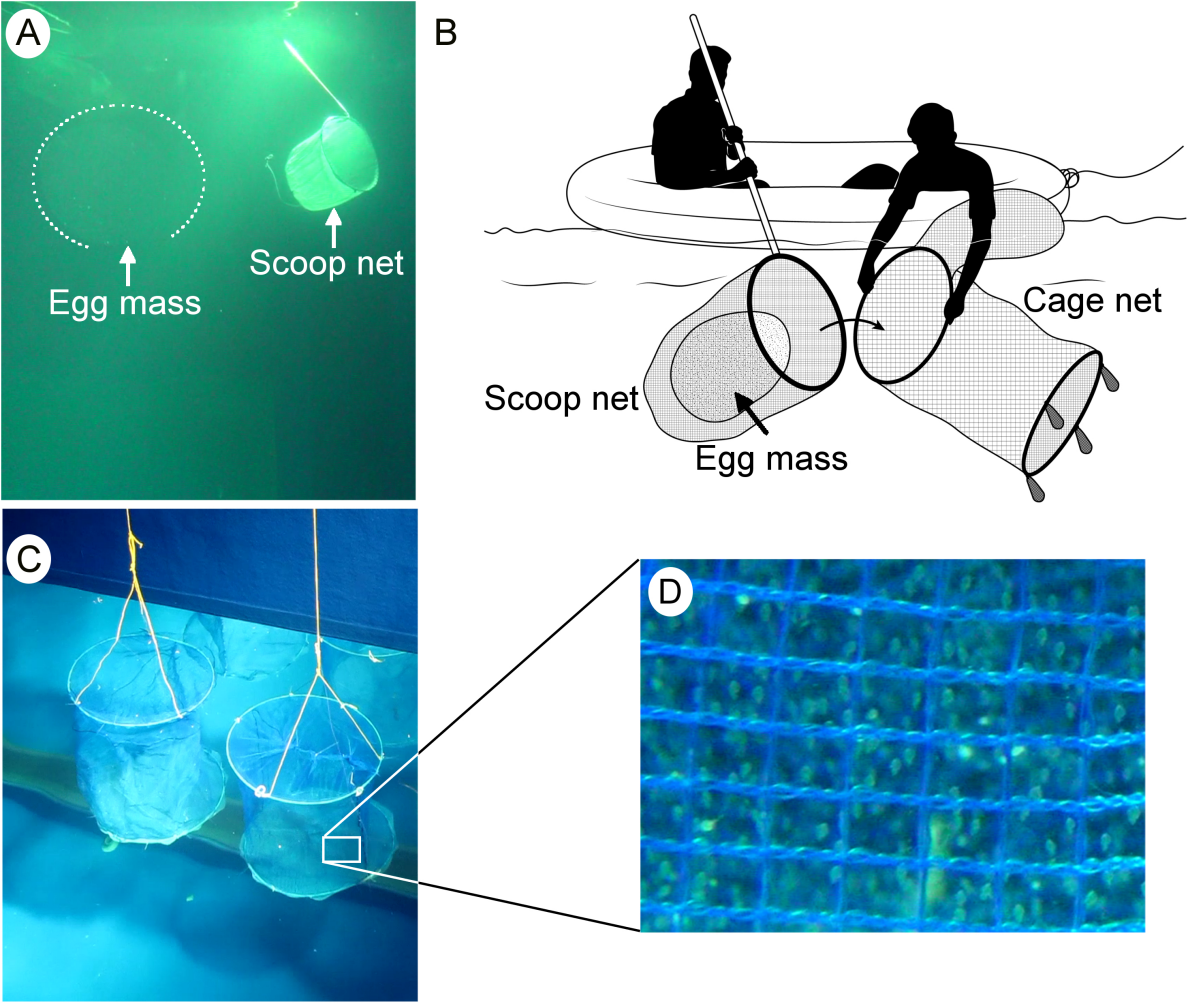
Collection and maintenance of egg masses in a cage net. (A) View of an egg mass and scoop net in the water column of the experimental tank. (B) Transfer of an egg mass to a cage net after collection with a scoop net. (C) Cage nets holding egg masses hanging near the observation window. (D) Enlarged view of a portion of the cage net showing developing embryos (as viewed through the observation window).

Egg masses constantly floated in the water column, inhibiting direct comprehensive observations. Thus, it was necessary to hold the egg mass in the water column to complete the investigation. Our previous attempt [13] of wrapping the egg masses with a gill net was partly successful, but altered the size and shape of the egg masses. In the present study, we employed appropriate cage nets that allowed the egg masses (<80 cm diameter) to be held, while facilitating the exchange of water and permitting convenient surveillance through the observation windows (Fig 2). The cage consisted of three basic components, the framework, weights, and the net. The framework consisted of two standard circular metal rods (diameter = ~1 m) with four weights (1 kg each) that were attached to one ring to maintain a vertical position in the water column. Two types of nets with varying mesh size were used to construct the cage net. The top (lid) and bottom of the cage were fitted with a fine net (mesh size = 0.2 mm). This net was stiffer to provide a flat surface to the top and bottom of the cage. The smaller mesh size also prevented any leakage/escape of egg masses through the cage bottom where they rest. The walls of the cage were fenced with a slightly larger mesh size (0.4 mm), so as to clearly view the fate of egg mass jelly and developing embryos.

Shortly (within 3 h) after spawning, the egg masses were measured, and appropriate (<80 cm diameter) sized egg masses (n = 11 individual egg masses) were transferred to the cage net. Transferal of the fragile gelatinous egg mass to the cage net required special care. Once the egg mass was located, two people were positioned onboard an inflatable boat in the tank. One person was assigned to collect the egg mass using a scoop net (mouth diameter, 1 m; mesh size, 1 cm), while the second person held the cage net to help with the transfer of the egg mass (Fig 2A and 2B). A scoop net was slowly placed in the water, and its mouth was moved carefully toward the egg mass. The egg mass constantly drifted along with the water current; thus, until collection (scooping), the position of the boat was maneuvered to correspond with that of the egg mass using ropes. Because the view of the egg mass from above the water (from the boat) was not always clear, one person was positioned in front of the observation window to communicate (using a walkie-talkie) the position of the egg mass in the water column, second by second. Once the egg mass was completely engulfed by the scoop net, the net was gently and slowly lifted upwards. Much care was taken to avoid breaking the egg mass or allowing it to escape from the net. When the egg mass reached the sub-surface, the second person held the cage net parallel to it, while it remained completely immersed under water (Fig 2B). Then, the egg mass was gently transferred from the scoop net to the cage net taking special care not to expose the egg mass to the air. The top (lid) of the cage was sealed after completely placing the egg mass in the cage. Once sealed, ropes were tied to the cage and it was hung in front of the observation window (Fig 2C, ~0.5 m above the bottom).

### Physical properties

The density and viscosity of the egg masses were analyzed. The outer nidamental gland jelly and inner mucous matrix were examined separately. To collect the outer jelly, the egg masses (n = 3) were first removed from the tank. Then, the dense outer jelly was isolated using a small scoop net (the inner mucous matrix and water escaped through the mesh). The inner mucous matrix was collected by inserting a pipette inside the intact egg masses in the tank.

For liquid, density (*ρ*) is determined from mass (m) and volume (v):

ρ=mv (1)

Viscosity measurements were made using a falling-sphere viscometer [35]. It involves using the time of free-fall of an object under the influence of gravity through the fluid of interest to determine viscosity. The principle of the falling-sphere is based on Stoke’s law. For a sphere of radius *a*, falling through distance *L*, the viscosity of the jelly (η) is given as follows:

$$\eta =\frac{2a^{2}(\rho_{s}-\rho_{j})gt}{9L}$$ (2)

where *ρ*_*s*_ is the density of the sphere, *ρ*_*j*_ is the density of jelly, *g* is the acceleration due to gravity (981 cm s^−1^), and *t* is the fall time. All measurements were made at room temperature (20°C).

### Video and image analysis

The fate of the egg mass jelly and embryo development were recorded by video using fixed and handheld Sony HDR-CX590V handy cams. All video footage was annotated, reviewed, and analyzed. Selected sequences from the videos (30 frames s^−1^) were captured with Adobe Premiere, and exported as frames into ImageJ (http://imagej.nih.gov/ij/) for detailed observation.

### Vertical profile of the spawning ground

The favorable temperature range for the development of *T*. *pacificus* paralarvae is between 18 and 24°C, particularly 19.5–23°C [28,29]. To determine how this temperature range correlates with the hypothesized settling depths (pycnocline) of the egg masses, we visualized the vertical profile of the spawning ground of *T*. *pacificus* during one spawning period (October 2015, autumn cohort). Monthly oceanographic data (temperature, density, and salinity) for two representative points (33.0°N 129.0°E and 31.5°N 126.0°E) at the spawning ground (Tsushima Strait, Japan) were obtained from the Global Ocean Data Assimilation System (GODAS), which was developed at the National Centers for Environmental Prediction (cfs.ncep.noaa.gov/cfs/godas/monthly).

## Results

### Egg masses

A total of 21 egg masses were spawned by 9 females over 21 days in the tank. The diameter of the egg masses ranged from 20 to 120 cm. For more information on the statistics of the spawned egg masses, see Puneeta et al. [34]. The gelatinous fragments from the initially spawned broken egg masses collected by the SCUBA diver were devoid of any eggs. These fragments were thick, indicating that they were part of the outer jelly of the egg masses. Portions of the fragments remained in the tank for about 5-days, before completely dissolving in the water.

**Fig 3:**
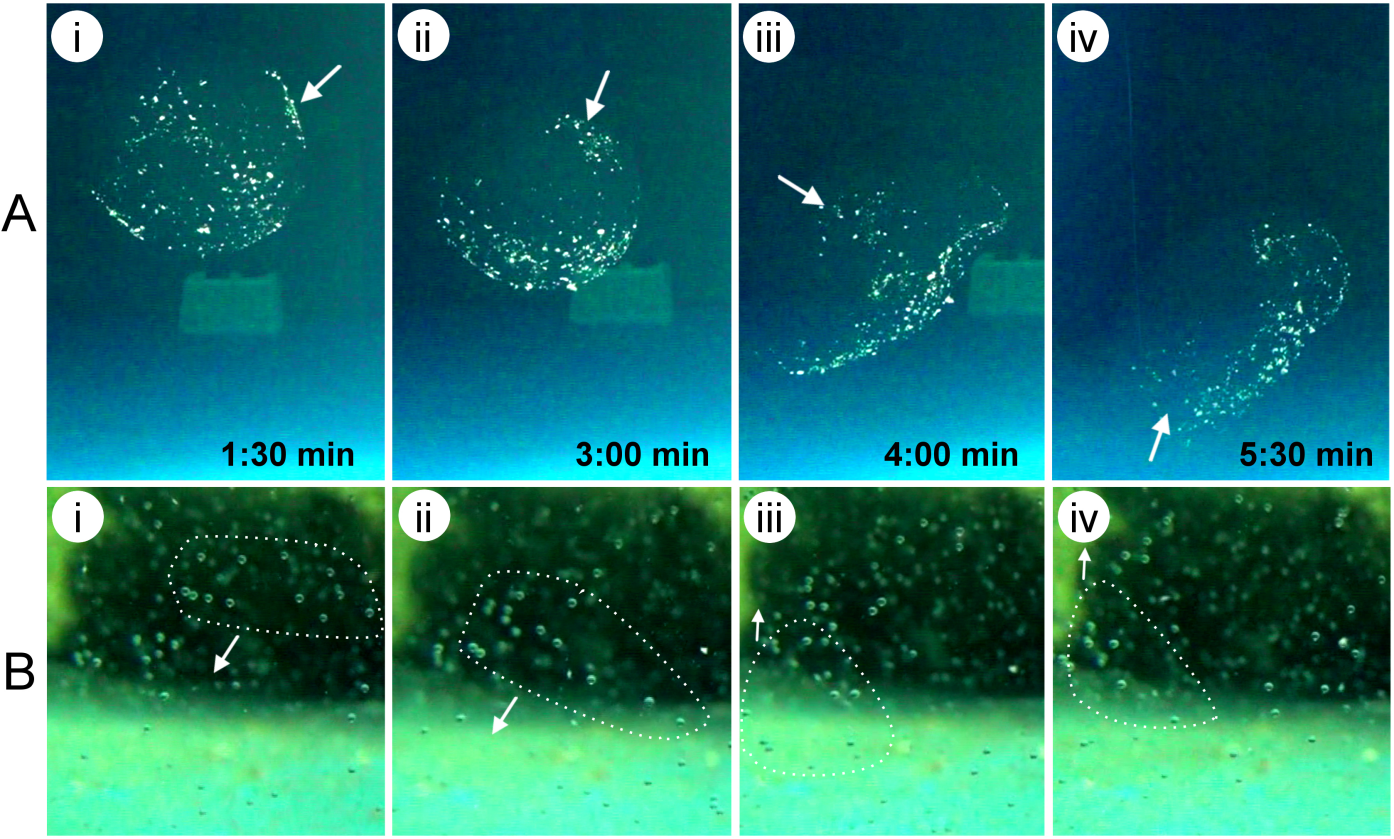
Drifting and undulation behavior of an egg mass. (*lane* A) The egg mass displays rotational behavior while drifting freely in the middle depth of the tank. The numerous white dots visible on the egg mass are debris from the water column. This deposited debris enhanced the visibility of the egg mass, which was otherwise very transparent. (*lane* B) Undulation of the embryos embedded within interior jelly matrix of egg mass. Arrows indicate the direction of movement.

Complete and unbroken egg masses freely floated in the mid-water column of the tank along with the gentle current (Fig 3A). In many instances, the whole egg mass structure showed extensive flexibility. It could twist and change shape according to the limitations it came across inside the tank, and returned to a normal spherical shape in a spacious environment. The egg mass almost transformed to a concave vase shape when pressed on either side by two other egg masses. The egg masses could completely roll over the rope (of the hanging cage net) without being broken. When pressed against the tank walls, the outer jelly operated like a balloon, jerking back to regain its shape.

**Fig 4:**
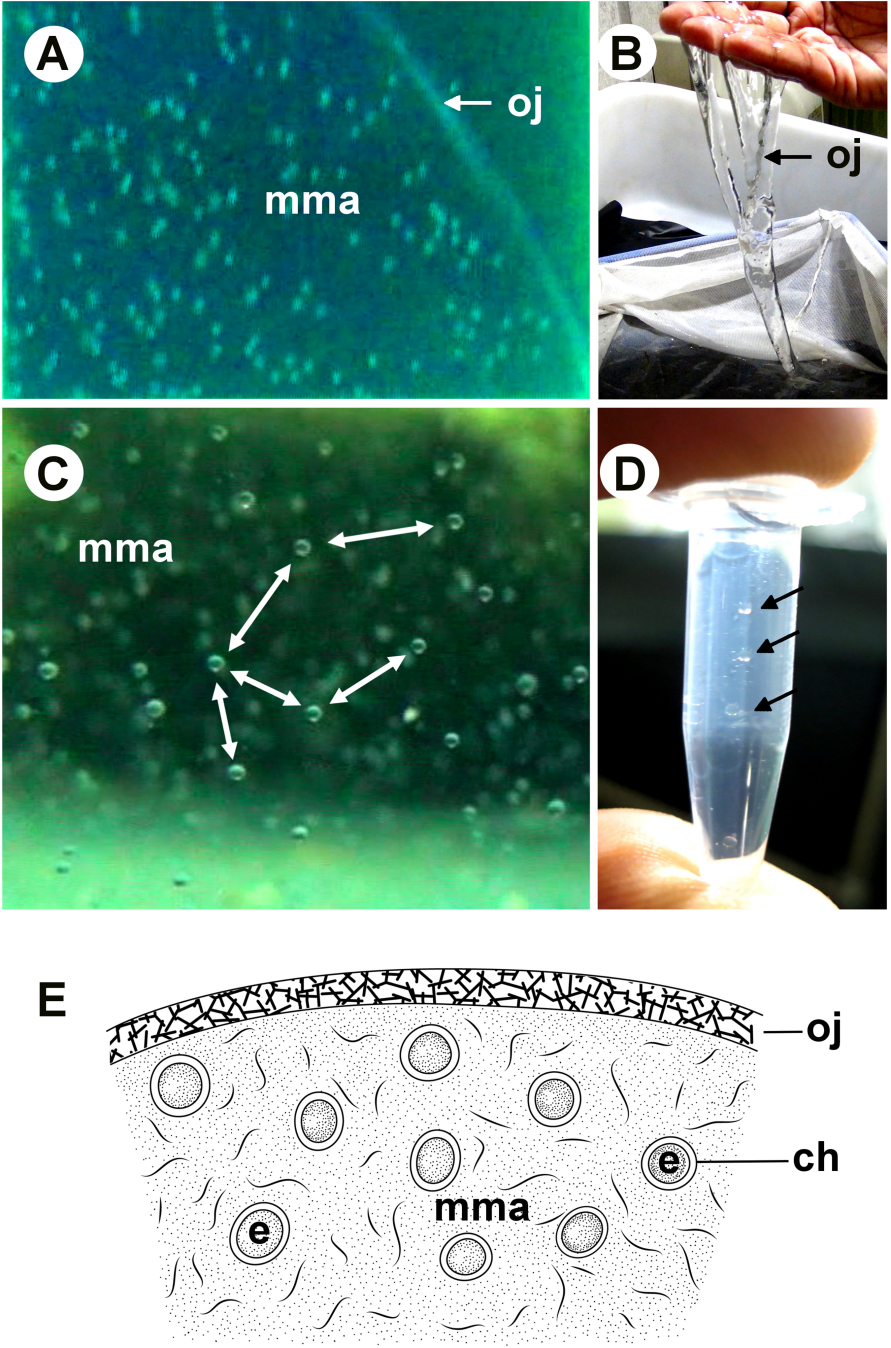
Egg mass structure. (A) Enlarged view of the side of an egg mass showing the outer jelly and internal mucous matrix. (B) Viscous nature of the outer jelly. (C) Arrangement of embryos within the egg mass in the internal mucous matrix. (D) Internal mucous matrix pipetted out showing the still floating embryos. Arrows show embryos. (E) Schematic of a longitudinal section of the light microscopic structure of a hypothetical egg mass. ch, chorion; mma, mucous matrix; oj, outer jelly.

All of the egg masses contained fertilized eggs. The developing embryos inside each egg mass displayed an undulating pattern that corresponded to the movement of the egg mass (Fig 3B); however, the embryos never touched each other. Video and image analysis clearly distinguished a thick outer jelly in the intact egg mass (Fig 4A). When separated, the egg mass was highly viscous but not sticky, and tended to flow unbroken (Fig 4B). The eggs/embryos were homogenously embedded inside the internal mucous matrix, with variable inter-egg space, but never touched each other (Fig 4C). The matrix acted as a medium to keep the embryo enclosed in the chorion afloat. Eggs/embryos that were pipetted out of the internal mucous matrix of the egg mass maintained a similar character, suspending away from each other (Fig 4D).

### Fate of egg masses over time

**Fig 5:**
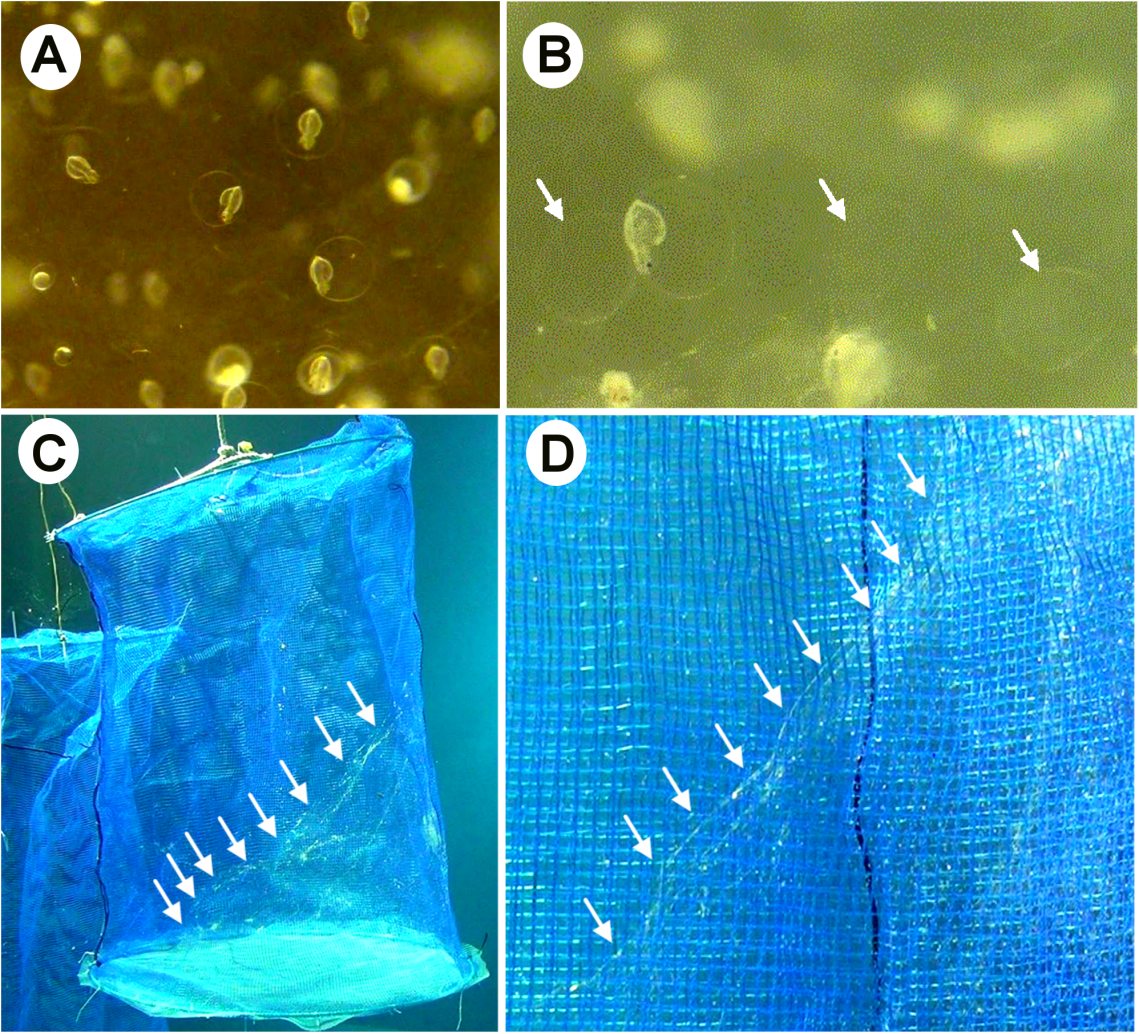
Fate of egg mass jelly. (A) Developing embryos inside the chorion within an egg mass. (B) After the embryos hatched, the empty chorion remained inside the egg mass. (C,D) Cobweb-like appearance indicating the disintegration of the gelatinous matrix of the egg mass. (D) Enlarged view of the disintegrated egg mass jelly from panel C. The jelly dissolved completely within ~24 h of embryos hatching. Arrows show the empty chorions in panels A & B, and indicate egg mass boundaries in panels C & D.

We were able to record the fate of embryos and egg mass jelly in close detail through observation of the egg masses held in the cage net. The egg masses were completely transparent after the first and second day of spawning. Fertilized eggs and embryos could be clearly seen through the outer jelly of the egg mass. At days 3–4, the egg mass was whiter, and the chorion surrounding the developing embryo became clearly visible (Fig 5A and 5B). As the embryo neared hatching (5–7 days), the outer jelly began to disintegrate and lost its shape, partially collapsing inside the cage (Fig 5C and 5D). Once the paralarvae hatched out the chorion, the fragments of disintegrated egg mass remained intact in the cage net for 2–3 h (Fig 5B).

There was a uniform trend in the hatching process, with the embryos that were distributed in the periphery hatching first (Day 5), followed by the innermost embryos (Day 6). Once all of the embryos had hatched, the egg mass continued to disintegrate, eventually disappearing completely. The largest egg mass (diameter: 120 cm) took 7 days to disintegrate, whereas intermediate sized egg masses (~60–80 cm) took 5–6 days at 19°C. The smallest egg mass (~40–50 cm) completely disintegrated on the fifth day.

### Physical properties and vertical profile of the spawning ground

**Fig 6:**
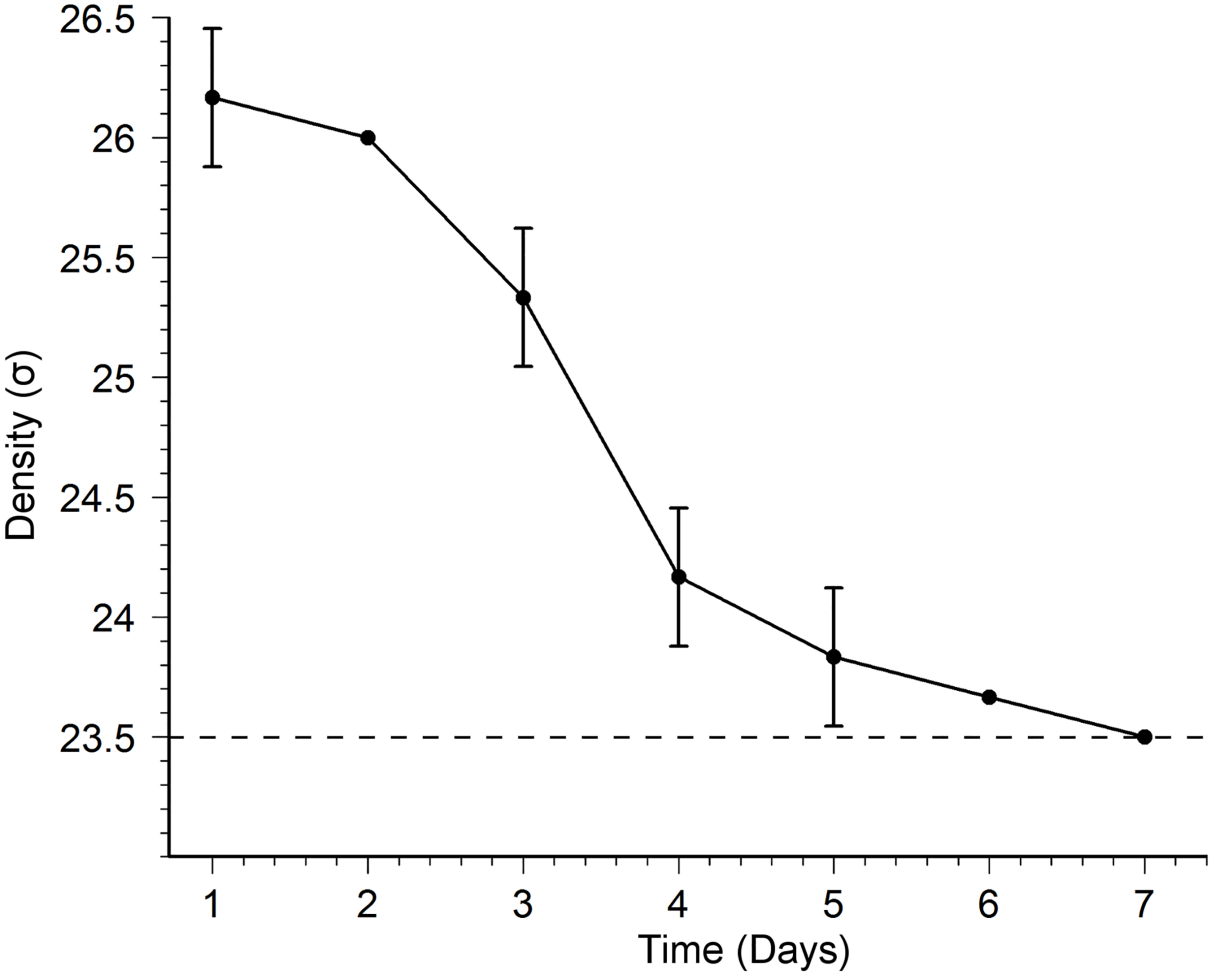
Changes to the density of the outer jelly through time. Solid circles are the mean values. Vertical bars are ±SD ranges. Dashed line denotes the density value of the surrounding seawater.

The visual appearance of the outer jelly was that of a highly dense viscous liquid (Fig 4B). The density of the outer jelly (*ρ*_*j*_) decreased from 1.0262 g cm^−3^ (σ = 26.2 kg m^−3^) on day 1 to 1.0235 g cm^−3^ (σ = 23.5 kg m^−3^) on day 7 (Fig 6). The day 7 value was similar to that of the seawater in the tank (σ = 23.5 kg m^−3^). Thus, the fresh jelly was 2.7 σ units higher than the seawater from which it was formed. The viscosity of the outer jelly collected on same day as spawning was 4.905 Pa s. On subsequent days, the viscosity of the jelly started to noticeably decline, completely losing its viscous nature on day 5, when it behaved like normal seawater. The viscosity of the inner jelly was similar to that of the seawater from day 1.

**Fig 7:**
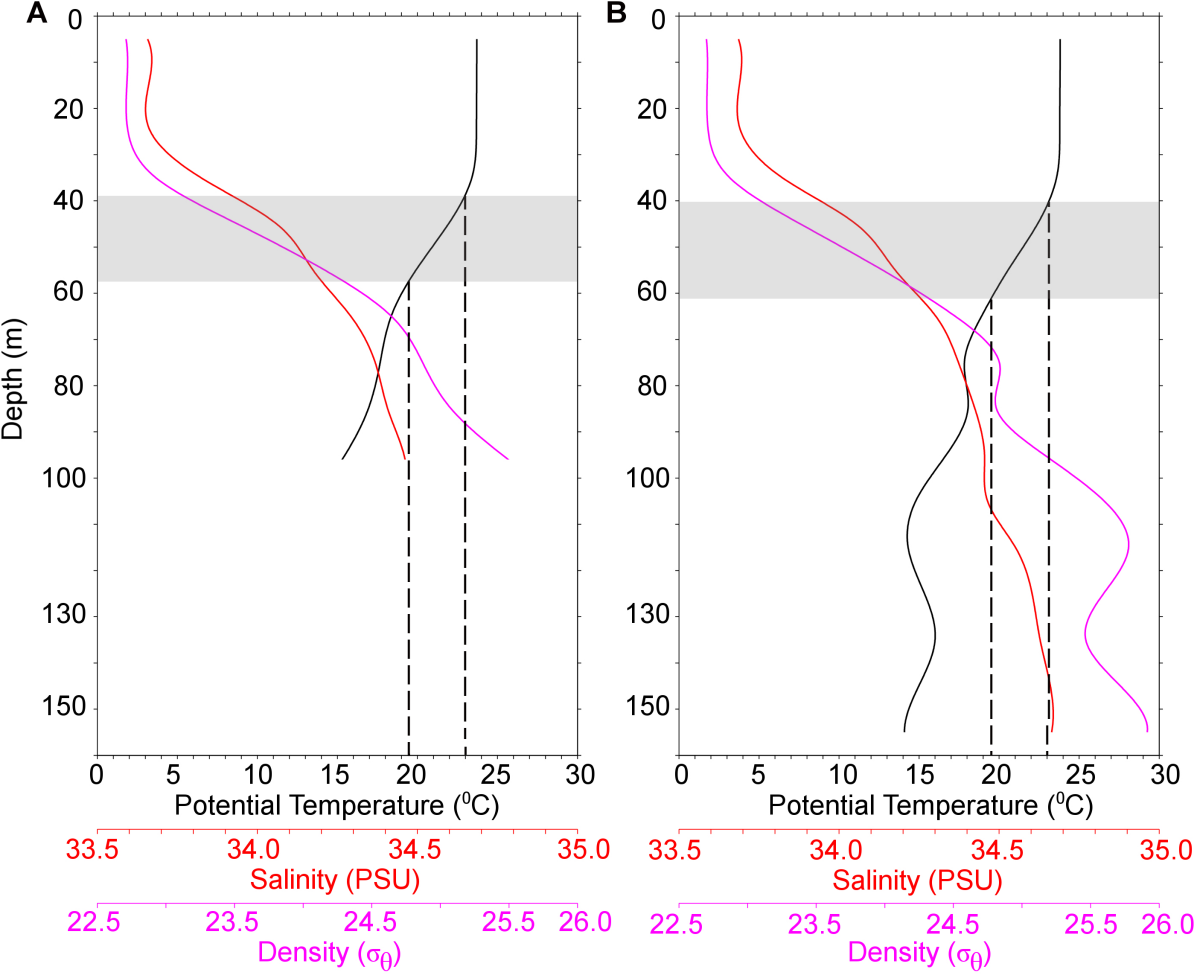
Vertical profiles of temperature (°C), salinity (PSU), and density (σ_θ_) at two locations in the Japanese flying squid (autumn cohort) spawning ground. (A) Bottom depth 95 m (33.0°N 129.0°E). (B) Bottom depth 155 m (31.5°N 126.0°E). In both A & B, the favorable temperature range (19.5–23°C) [29,32] for *T*. *pacificus* hatchling survival coincided with the depth range (≈40–60 m, shaded region) where the density and temperature gradient increased (i.e., pycnocline and thermocline).

The vertical profiles of two locations at the spawning ground (Tsushima Strait) of *T*. *pacificus* are shown in Fig 7. The thermocline and pycnocline were distributed at ≈40–60 m. Irrespective of differences in the bottom depth of the two locations (95 and 155 m), the favorable temperature range (19.5–23°C) [29,32] for the survival and swimming activity of hatchling *T*. *pacificus* coincided with the depth of the pycnocline/thermocline layer.

## Discussion

### Structure of the egg masses

The jelly substance (fragments) that was collected from the broken egg masses was devoid of eggs, indicating that, once broken, the eggs leaked out into the surrounding water. Eggs outside of the egg mass either develop abnormally or stop developing altogether [13,14]. The fragments were thick and viscous in nature, suggesting that they were part of the outer jelly of the egg mass. Thus, intact, complete spherical (or near to spherical) egg mass structures are required to support the arrangement of eggs and ensure the survival of embryos. However, nidamental gland jelly is only required for the survival of ommastrephid embryos under natural conditions. In artificial fertilization experiments, healthy paralarvae are produced without nidamental gland secretions [22, 31, 36–40]. The embryos of oegopsid squids caught in the plankton net also later developed in an incubator and hatched without the gelatinous material surrounding it [2].

Because the egg masses of *T*. *pacificus* are freely spawned in the water column, and are then abandoned by the parent, protective layers (for protection against mechanical damage, infection, predation, and, possibly, osmotic stress) must be provided for the developing offspring to survive [41]. Nutritive layers are necessary to ensure that enough energy is present for the embryos to finish normal development until hatching [41]. While various hypotheses exist [4,5] regarding egg mass formation and settling depth, information about their fine-scale structure and properties remains limited. This information is necessary to fill the remaining gaps of our basic knowledge on this critical period of the life cycle of *T*. *pacificus*. It is not possible to visualize the actual structure of the different layers constituting the egg mass by taking a cross section of it because it is watery and non-sliceable. Based on our results and available information from the published literature [4,13,20], we hypothesize that the egg mass has a thick outer layer made from nidamental gland secretion and an internal mucous matrix (mma) derived from a mucosubstance originating from the oviducal gland secretion (Fig 4E). The outer jelly could be multi-layered because Kimura et al. [20] obtained components of different biochemical natures after analyzing the egg mass of *T*. *pacificus*. Chemically, the surface layer is derived from the water-soluble fraction of the nidamental mucosubstance, while the fibrils within the mass (Fig 4E) are derived from the insoluble fraction of the mucosubstance [20]. The presence of fibrils in the mma could help maintain the distance between the eggs, as well as regulate their homogenous distribution inside the egg mass. The mma was very flexible in accommodating the growing embryo. As the embryo developed, the chorion, which is the protective coating of the embryo, expanded to be twice as long (~2.3 mm) as the embryo (~1 mm); consequently, the chorion occupied more space inside the egg mass than when it was first spawned. This increase in chorion size did not affect the overall size of the egg mass, but was adjusted for by a decline in the inter-embryonic space.

The movement of the egg mass and the corresponding undulation of the embryos might help mediate the distribution of oxygen within the internal mucous matrix. An egg mass of 80 cm diameter was packed with ~200,000 embryos from the periphery to the center. The embryos develop fast (hatching on about day 5), further reducing the space within the egg mass as they increase in size. In this congested situation, embryos developing in the center might face a deficiency in the oxygen supply. Giorgi and Congleton [42] reported the retarded development and death of central embryos, along with reduced interstitial oxygen concentration, within the large masses of adherent eggs of ling cod at field sites with low current velocities. The delayed development of embryos in the central positions of egg masses of gastropod mollusks is usually linked to reduced metabolic rates, due to low ambient pO_2_ [43]. To overcome this hypoxia problem in the center of the egg mass, embryos in the periphery and center develop separately in some gastropod mollusks [44], with embryos at the periphery hatching first. Because all *T*. *pacificus* hatch within a short time frame (10–20 h), some other mechanism, like undulation, probably enhances the rate of oxygen exchange. However, in the *T*. *pacificus* egg mass, hatching is also ordered from the periphery to the center. Thus, to understand the advantage of this pattern, further investigation is required.

### Properties of the egg mass

The technique that we developed here using a cage net to suspend the egg mass, allowed the shape of the egg mass to be retained (except for the bottom resting portion), while also allowing us to obtain a close-up view of the fate of the egg mass. The outer jelly of the egg mass appeared to be very stiff and viscous, because mollusk gels are composed of protein-polysaccharide complexes (glycoproteins or proteoglycans) that have a structure made up of a cross-linked network of proteins [45,46]. Resistance to any deformation (stiffness and viscosity) depends on the number and strength of the cross-links [46]. High viscosity (4.905 Pa s) helped the outer jelly to maintain the shape of the egg mass inside the cage, and might also help to protect it against adverse conditions in the sea. For example, the sensitivity of the egg masses to the water current in the tank indicated that weak currents can carry egg masses away from the spawning site without breaking them. However, strong currents could destroy the egg masses. The threshold of egg mass resistance to currents might determine the extent of horizontal dispersal.

To understand the vertical distribution of floating egg masses in the sea, we must understand the physical properties of the egg mass and combine this information with distribution surveys [33,47]. Considering the lack of available direct data on the distribution of *T*. *pacificus* egg masses in the wild [48], data on the physical properties from laboratory studies are the only available source from which to derive assumptions on their vertical distribution. Comparison of this data to that inferred from the spawning ground in the context of the “reproductive hypothesis” of *T*. *pacificus* could provide important insights about their distribution. In accordance with this hypothesis, the density of the egg mass is the key factor determining the settling depth of *T*. *pacificus* egg masses in/above the pycnocline layer [1,5]. Components contributing to the overall density of the egg mass are the density of the outer jelly and the specific gravity of individual eggs. We found that the density of the outer jelly was 2.7 σ units higher than the seawater in the tank, whereas the density of the internal mucous matrix was similar to that of the seawater from which it was formed. The resultant overall density of the egg mass is slightly denser than the seawater, because the outer jelly constitutes only a minor fraction of the egg mass component, with the specific gravity of individual eggs being is about 1.10 [9]. This difference results in the egg mass having very slight negative buoyancy relative to the water in which it was formed [9]. As a result, the egg mass sinks slowly until it reaches a depth at which the density is in equilibrium with the surrounding seawater. In the spawning ground (Tsushima Strait, Fig 7) of *T*. *pacificus*, variation in the density is negligible until ~30 m deep (probably mixed layer), and sharply increases below 30 m (pycnocline layer). The temperature in the top layers of the pycnocline (~40 m) is within the optimum temperature ranges suitable for *T*. *pacificus* embryonic development. The density at this depth is around 0.4 σ_θ_ units higher than the surface water. This density might be too great for eggs to pass; thus, it might create a strict boundary in the vertical distribution of egg mass. In conclusion, *T*. *pacificus* egg masses might retain their location in the water column by floating at the interface between water layers of slightly different densities, which happen to be above the pycnocline layer (actual depth varies seasonally/annually) in the Tsushima Strait between Korea and Japan.
